# Limited contributions of plant pathogens to density‐dependent seedling mortality of mast fruiting Bornean trees

**DOI:** 10.1002/ece3.6906

**Published:** 2020-10-25

**Authors:** Patrick G. Cannon, Michael J. O’Brien, Kalsum M. Yusah, David P. Edwards, Robert P. Freckleton

**Affiliations:** ^1^ Department of Animal and Plant Sciences The University of Sheffield Sheffield UK; ^2^ Área de Biodiversidad y Conservación Universidad Rey Juan Carlos Móstoles Spain; ^3^ Danum Valley Field Centre South East Asian Rainforest Research Partnership (SEARRP) Lahad Datu Sabah Malaysia; ^4^ Institute for Tropical Biology and Conservation Universiti Malaysia Sabah Kota Kinabalu Sabah Malaysia

**Keywords:** competitive exclusion, conspecifics, density dependence, *Dipterocarpaceae*, fungal pathogens, Janzen‐Connell

## Abstract

Fungal pathogens are implicated in driving tropical plant diversity by facilitating strong, negative density‐dependent mortality of conspecific seedlings (C‐NDD). Assessment of the role of fungal pathogens in mediating coexistence derives from relatively few tree species and predominantly the Neotropics, limiting our understanding of their role in maintaining hyper‐diversity in many tropical forests. A key question is whether fungal pathogen‐mediated C‐NDD seedling mortality is ubiquitous across diverse plant communities. Using a manipulative shadehouse experiment, we tested the role of fungal pathogens in mediating C‐NDD seedling mortality of eight mast fruiting Bornean trees, typical of the species‐rich forests of South East Asia. We demonstrate species‐specific responses of seedlings to fungicide and density treatments, generating weak negative density‐dependent mortality. Overall seedling mortality was low and likely insufficient to promote overall community diversity. Although conducted in the same way as previous studies, we find little evidence that fungal pathogens play a substantial role in determining patterns of seedling mortality in a SE Asian mast fruiting forest, questioning our understanding of how Janzen‐Connell mechanisms structure the plant communities of this globally important forest type.

## Graphical abstract

Assessment of the role of fungal pathogens in mediating coexistence derives from relatively few tree species and predominantly the Neotropics, limiting our understanding of their role in maintaining hyper‐diversity in many tropical forests. Using a manipulative shadehouse experiment, we find little evidence that fungal pathogens play a substantial role in determining patterns of seedling mortality of eight mast fruiting Bornean trees. Our results starkly contrast those of similar neotropical studies, questioning our understanding of how Janzen–Connell mechanisms structure the plant communities of this globally important forest type.

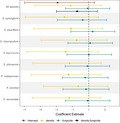

## 1 INTRODUCTION

Tropical regions contain ~53,000 tree species (Slik et al., 2015), with as many as 1,200 species coexisting in just 52 hectares of moist tropical forest (Lee et al., 2002). Understanding the mechanisms that maintain such extraordinary plant diversity remains a central question in ecology. After nearly 50 years of study, the Janzen–Connell (JC) hypothesis (Connell, 1971; Janzen, 1970) remains the most widely posited explanation for preventing competitive exclusion. Central to JC‐theory are the effects of conspecific negative density‐dependence (C‐NDD), whereby high densities of conspecific recruits are more likely to succumb to specialist natural enemies, such as insect herbivores (Jia et al., 2020), and soil and fungal pathogens (Bagchi et al., 2010; Mangan et al., 2010). Evidence of C‐NDD has been reported globally (Carson et al., 2008; Comita et al., 2014), with a variety of observational, experimental, and modeling approaches linking pathogen‐induced C‐NDD to the enhancement of plant community diversity (Bagchi et al., 2011; Harms et al., 2000; Krishnadas et al., 2018). Density dependence, therefore, represents an important regulator in the maintenance and structuring of hyper‐diverse plant communities.

Although seed predators and herbivores alter seedling recruitment and plant community composition in both the New‐ and Old‐World (Granados et al., 2017; Villar et al., 2019), whether their effects are sufficient to drive C‐NDD remains unclear (Hautier et al., 2010; Kurten & Carson, 2015). In contrast, host‐specific impacts of fungal pathogens responsible for driving C‐NDD are well‐documented (Augspurger & Kelly, 1984; Freckleton & Lewis, 2006), with fungal pathogens the most important seedling enemies (Sarmiento et al., 2017). However, studies demonstrating fungal contributions to the Janzen–Connell mechanism are limited to a small number of tree species and systems, with pervasive neotropical bias (Bagchi et al., 2011; Comita et al., 2014). Examination of the C‐NDD literature identified 17 studies manipulating seed or seedling densities of tropical trees, of which only five experimentally tested the impacts of fungal pathogens on just 12 species, with the majority of studies focusing on the neotropics (65%; Table S1). Most notably, representation of fungal‐induced C‐NDD is dominated by a single common neotropical species—*Pleradenophora longicuspis* (Standl.) Esser.—demonstrating intense overcompensating density‐dependence and causing up to 100% seedling mortality (Bagchi et al., 2010; Bell et al., 2006; Swinfield et al., 2012).

The strength and presence of reported C‐NDD vary considerably between studies (Bagchi et al., 2011; Harms et al., 2000). Recent work has highlighted the pervasive use and mismanagement of error‐prone predictors when quantifying C‐NDD (Detto et al., 2019), resulting in the erroneous detection and overestimation of the strength of density dependence in many studies. Observational studies are particularly prone to such errors in comparison to manipulative experiments, highlighting the importance of considering methodological approach in addition to ecological context when examining evidence of C‐NDD. Bagchi et al. (2014) for example used a design that explicitly accounts for census error, a known bias in measurement of NDD (Freckleton Freckleton & Lewis, 2006), when estimating forest‐wide impacts.

Notwithstanding methodological issues, variation in the strength of C‐NDD likely reflects differences among species (Carson et al., 2008; Comita et al., 2014), with negative effects of conspecific density posited to more strongly influence rare rather than locally common species (Johnson et al., 2012; Mangan et al., 2010). Species life‐history strategies also affect the strength of C‐NDD (Jia et al., 2020), with trade‐offs between axes of growth and defence, at least partly, governing variation in species susceptibility of pathogens (Milici et al., 2020; Spear et al., 2015), potentially influencing seedling recruitment patterns. Shade‐tolerant species are more susceptible to fungal pathogens (Kobe & Vriesendorp, 2011; McCarthy‐Neumann & Kobe, 2008), particularly in fragmented forests (Krishnadas et al., 2018). Interactions between abiotic gradients and species life‐histories may also work to maintain and enhance plant diversity via C‐NDD and other mechanisms, including by heightening niche partitioning along light gradients (McCarthy‐Neumann & Kobe, 2008) and increasing conspecific seedling mortality under higher precipitation (Milici et al., 2020). It is, therefore, important that future studies investigate a wider suite of species (Bagchi et al., 2010; Bell et al., 2006; Carson et al., 2008; Comita et al., 2014; McCarthy‐Neumann & Kobe, 2008; Pillay et al., 2018; Segnitz et al., 2020) encompassing variation in species life‐history strategies and local abundance, particularly in tropical forests where the majority of species are locally rare.

Forests of the Indo‐pacific region, in particular South‐east Asia, are among the most species rich on Earth (Sullivan et al., 2017). Dominated by trees of the *Dipterocarpaceae* (Saner et al., 2012), reproduction occurs in supra‐annual community‐wide mast fruiting events, with hundreds of tree species fruiting synchronously (Curran & Leighton, 2000; Janzen, 2004). Such events are preceded by years of limited or no fruit fall and are suggested to have evolved to satiate a reduced number of seed predators (Curran & Leighton, 2000; Visser et al., 2011). Masting‐induced predator satiation decreases mortality in areas of high density, directly undermining C‐NDD. In addition, although common seed predators such as bearded pig (*Sus barbatus*) are a significant source of seed and seedling mortality within mast‐fruiting forests (Granados et al., 2017), such predation pressure is not host‐specific and consequently is incapable of driving C‐NDD (Freckleton & Lewis, 2006). It is, therefore, hypothesized that determinants of the Janzen‐Connell mechanism, including density‐dependence, may be substantially weaker or entirely absent among mast‐fruiting species (Bagchi et al., 2011; Comita et al., 2014; Janzen, 1970). In contrast, the reservoir of host‐specific fungal pathogens will be greatest in soils under adult trees and greater still in areas of high adult density (Liang et al., 2016). During mast events, these areas will also experience the greatest densities of seeds and young seedlings with the potential to exert strong NDD mortality of conspecific seedlings.

Despite comprising ~48% of tropical tree species (Slik et al., 2015), there are just three studies manipulating seed or seedling densities from the Asian tropics (Table S1). Only one of these studies investigated the impacts of fungal pathogens (Krishnadas & Comita, 2018), finding significant pathogen‐induced mortality of three of the four focal species but that these effects were independent of density. The two remaining studies from tropical Asia did not experimentally test the contributions of different natural enemies for one (Lott et al., 1995) or two (Takeuchi & Nakashizuka, 2007) species, respectively. Fungal pathogens are widely posited to represent a hugely influential demographic force near ubiquitously across forest systems despite limited evidence from many tropical forests. Determining whether C‐NDD is present across species communities and other forest systems remains a key unanswered question.

Although more numerous than experimental approaches, observational studies examining C‐NDD in Asian tropical forests demonstrate contrasting evidence for C‐NDD. Some provide support for C‐NDD (Bagchi et al., 2011; Pillay et al., 2018), while others have found limited evidence of density‐dependent effects (Itoh et al., 1995; Maycock et al., 2005), or only at specific life‐stages (Blundell & Peart, 2004). Additionally, in masting systems, many studies utilize partial fruiting episodes (Harms et al., 2000; Maycock et al., 2005) when near‐complete seed and seedling mortality are likely unrepresentative of intense masting events when the vast majority of recruitment occurs (Curran & Leighton, 2000). To date, only a single study has experimentally tested for mechanisms underlying C‐NDD during an intense masting event (Pillay et al., 2018). Focusing on a single locally common species, Pillay et al., 2018 attributed a proportion of density‐dependent mortality to vertebrate seed predators and seedlings herbivores. Importantly, contributions of fungal pathogens to C‐NDD within mast fruiting forests and across multiple species have yet to be quantified.

We describe a manipulative shadehouse experiment using seedlings of eight species, from a lowland tropical forest in Sabah, Malaysian Borneo. During the 2019 mast fruiting event, we artificially manipulated seedling planting density and exclude fungal pathogens via the application of broad‐spectrum fungicides to assess fungal pathogen‐induced density‐dependent mortality across the seed‐to‐seedling transition. We test the predictions that: (a) despite the hypothesized influence of masting on the Janzen–Connell mechanism, seedlings will express C‐NDD mortality across species (Comita et al., 2014), finding greater mortality of individuals planted at higher density; (b) C‐NDD is driven by fungal pathogens, with limited mortality of individuals planted at either density when receiving fungicide treatments; and (c) the strength of fungal‐induced density‐dependence varies widely between species (Freckleton & Lewis, 2006), with rare species, comprising fewer stems in the surrounding forest, experiencing stronger C‐NDD mortality (McCarthy‐Neumann & Kobe, 2008). Our aim is to determine the role of fungal pathogens in mediating C‐NDD across our focal species, giving insight to their role in maintaining and structuring the hyper‐diverse plant communities of a globally important forest type (Edwards & Laurance, 2013; Sullivan et al., 2017).

## 2 MATERIALS AND METHODS

### 2.1 Study area

The experiment was conducted from the 31st August to 8th November 2019 in a single shadehouse at the Malua Field Station (MFS), Sabah, Malaysian Borneo (N05°05′20″ E117°38′32″; 102 m a.s.l.). Mean (*SD*) monthly precipitation at the site (averaged between August 2008 and December 2019) was 266.8 (105.7) mm. Across the duration of the study, mean (*SD*) monthly precipitation was 219.4 (71.3) mm (141.2 mm September, 280.9 mm October and 236.1 mm November), with mean ambient air temperature ranging from 21.8°C–35.7°C. Climatic data were recorded at the MFS weather station (available at; http://www.searrp.org/scientists/available‐data/). Shadehouse construction consisted of two sheets of rain permeable 70% shadecloth, creating a light environment with a mean (*SD*) of 9.34% (1.01) of full daylight photosynthetically active radiation (PAR), with means from five measurement locations across the shadehouse varying between 3.40% and 20.13%. Variability in the light environment was recorded using a quantum sensor (QS5 sensors; Delta‐T Devices, Burwell, Cambridge, UK), comparing simultaneous shadehouse readings to those from an unobstructed forest clearing. Ambient air temperature within the nursery averaged 24.8°C (0.07), with mean relative humidity 99.43% (0.11).

We collected seeds from mature individuals of eight lowland tree species during the 2019 community‐wide mast fruiting event, including six species of *Dipterocarpaceae*, one *Malvaceae* and one *Leguminosae* (Table S2, nomenclature standardized to that of *The Plant List*, V 1.1). Together, the selected species represent 2.32% of adult stems within neighboring primary forest (data from the 50‐ha Plot Project Danum Valley, part of the CTFS‐ForestGEO network (Anderson‐Teixeira et al., 2015)). Seeds of six species were collected from the surrounding logged forests of the 1 million ha Yayasan Sabah Forest Management Area (YSFMA), including the Ulu Segama, Taliwas and Malua Forest Reserves. Additionally, seeds of two species (*Scaphium macropodum,* Miq. and *Shorea pauciflora*, King) were collected from the nearby Danum Valley Conservation Area (DVCA). The DVCA is an 43,800 ha area of old‐growth forest, predominantly of *Dipterocarpaceae* (Saner et al., 2012). Forests of the YSFMA were selectively logged during the 1970s and 1980s, with much re‐logged 1999–2007. The majority of marketable hardwood stems over 40 cm d.b.h were removed, with extractions of ~113 and ~144m^3^/ha in once‐ and twice‐logged forest, respectively (Fisher et al., 2011).

### 2.2 Fungal exclusion and density‐dependence experiment

During the peak of the 2019 mast fruiting event (23rd–27th August 2019), we collected the topmost *c*. 15 cm of soil and humic matter from 12 once logged sites surrounding the Malua Field Station. All sites were located at least 50 m from the forest edge, with obvious signs of prior seed fall preceding soil collection in the majority of sites. Soil was sieved by hand, removing larger debris, roots, and seeds. Once processed, 364 circular pots (20 cm D × 20 cm H; 6.1 L V; 0.342 m^2^) were filled with *c*. 15 cm depth of unmixed soil, with each soil site replicated across treatments and species.

Seeds were germinated in optimal conditions using wet burlap sacks. Once germinated, viable seeds were randomly selected and planted in a uniform pattern at either a high or low density. Ten seeds were planted in High‐density pots and a single seed in Low‐density pots, corresponding to densities of 318 seeds/m^2^ and 31.8 seeds/m^2^, respectively. High‐density treatments were, by design, significantly higher than recorded in local seedling banks, with seed densities under Dipterocarp trees during the 2019 fruiting ranging from 1 to 183 m^‐2^ (O’Brien, Unpublished data). This was done to ensure that any effects of density (particularly over‐compensating density dependence) would be clearly evident or, conversely, that weak or undetectable effects of density would clearly indicate that such effects are ecologically unimportant.

Pots were arranged in blocks of four, at least 15 cm apart, with each pot receiving one of four treatment combinations: (a) High‐density/non‐fungicide, (b) High‐density/fungicide, (c) Low‐density/non‐fungicide, and (d) Low‐density/fungicide. Treatments were randomly assigned to pots with each treatment represented in each block (following Bell et al., 2006).

Within fungicide‐treated pots, seedlings were sprayed with a combination of Amotan 22.8SC^®^, a selective methalaxyl‐based systemic fungicide (Advansia Ltd, Malaysia, active ingredient: azoxystrobin), and Kencozeb M45^®^, a broad‐spectrum di‐thiocarbamate non‐systemic fungicide (Kenso corporation, Selangor, Malaysia, active ingredient: mancozeb). Active ingredients of both fungicides are frequently used to test fungal contributions to seedling mortality (Krishnadas et al., 2018; Szefer et al., 2020; Xu et al., 2015), providing protection against plant pathogenic fungi and oomycetes, with low toxicity to other soil biota and with minimal inhibitory effects on arbuscular mycorrhizal associations with tropical seedlings (Gripenberg et al., 2014). Fungicides were applied to seedling leaves, or to the surface of germinated seeds prior to leaf development, once every 10 days for 60 days (6 treatments) at the recommended concentration (Amotan22.8SC^®^; 0.05 ml/m^2^, Kencozeb M45^®^; 0.11 g/m^2^) using a hand mister, with 50 ml of solution per pot. The same volume of water was applied to non‐treated pots as a control. To prevent accidental treatment of control pots, a spray guard was used when applying fungicide. The number of seedling deaths in each pot was recorded every two days for the first month of the experiment, every four days for the second month, and once every 30 days thereafter. Any additional, newly germinated seedings were removed during the first two months of censusing.

After 60 days, we measured the height and diameter at soil of all remaining seedlings. Fungal pathogens are responsible for many damping‐off diseases, causing folia damage and mortality in young seedlings (Cohen & Coffey, 1986). To quantify fungal damage to leaves, we also recorded a measure of leaf damage, using an established four‐point ordinal scale (0 = no damage, 1 = 1%–25%, 2 = 26%–50%, 3 = 51%–75%, and 4 = 76%–100%).

### 2.3 Statistical analysis

We analyzed the proportion of planted seedlings surviving after 60 days using hierarchical linear models, assuming a binomial error distribution and logit‐link function. The model included seedling density, fungicide treatment, and the interaction between density and fungicide as fixed factors, owing to the fully balanced factorial experimental design. We included pot identity as a random intercept to account for spatial variation between replicates, and random uncorrelated slopes for fungus and density per species to account for interspecific variation in seedling responses to treatments. Model parameters were estimated within a hierarchical Bayesian framework using R package *brms* (Bürkner, 2017), sampling the model posterior using the NUTS algorithm within Stan software (version 2.17.050). The framework uses statistical shrinkage to produce robust estimates of species coefficients. Models converged without divergent transitions.

We extracted species‐level regression coefficients of density and fungicide effects from seedling survival models to determine the strength of C‐NDD effects on species survival. We use a simple ordinary least squares model to analyze the relationship between the strength of C‐NDD effects and species’ relative abundances from sample plots in the unlogged 50‐Hectare Plot Project, Danum Valley (Anderson‐Teixeira et al., 2015). We chose to use community data from an undisturbed site, rather than surrounding sites that provided seeds and soils, as this more accurately reflects true species’ abundances prior to logging activity. Note that these density data are independent of the experimental results.

We used ordinary least squares models (LM) to test the effects of density and fungicide treatments on seedling diameter, height, and leaf damage score. Data were modeled both collectively for all seedlings and for each species separately, including species, seedling density, fungicide treatment, and the interaction between density and fungicide as fixed factors. The level of independent replication was the pot. Analyses were carried out using R statistical software (version 3.5.2).

## 3 RESULTS

### 3.1 Seedling survival

**Figure 1:**
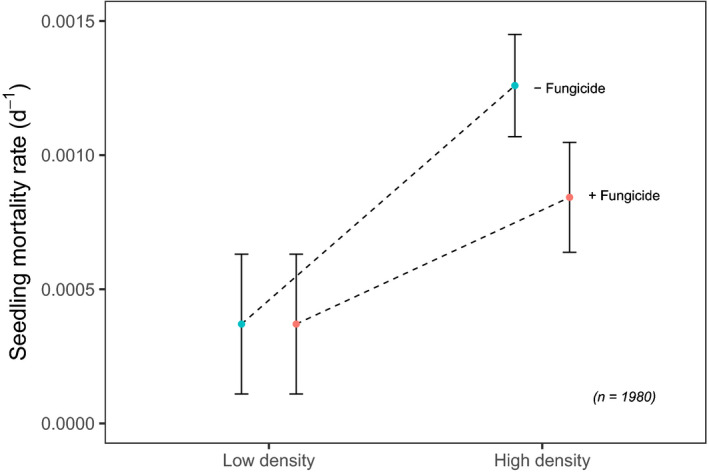
Daily seedling death rate between pots planted at high and low density and treated with (+) or without (‐) fungicide aggregated across all 8 species. Data points represent means of 90 replicates (*n* = 900 individuals for each high‐density treatment, and *n* = 90 for low‐density treatment) and error bars denote standard error

**Figure 2:**
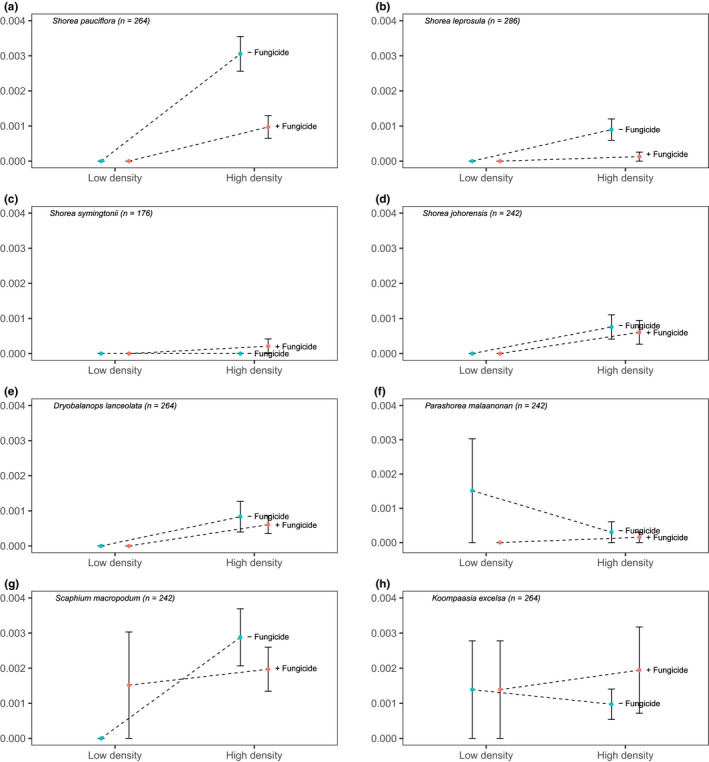
Daily seedling death rate between pots planted at high and low density and treated with (+) or without (‐) fungicide for each species: (a) *Shorea pauciflora*, (b) *Shorea leprosula*, (c) *Shorea symingtonii*, (d) *Shorea johorensis*, (e) *Dryobalanops lanceolata*, (f) *Parashorea malaanonan*, (g) *Scaphium macropodum*, and (h) *Koompasia exelsa*. Data points represent means of 8–13 replicates (*n* = 80–130 individuals for each high‐density treatment, and *n* = 8–13 for low‐density treatment) and error bars denote standard error

**Figure 3:**
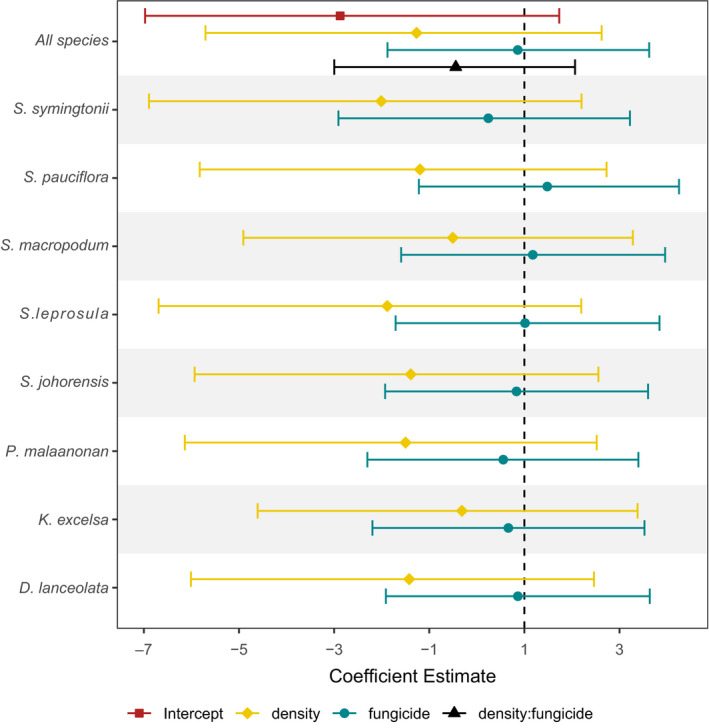
Overall and species‐level effects of planting density (high or low), fungicide treatment (+ or −) and the interaction between density and fungicide on seedling mortality for eight lowland tree species. Points represent the difference between the means of treatments and controls (i.e., the difference between high and low density, and fungicide and controls). Bars represent 95% Bayesian credible intervals of parameter estimates. Random uncorrelated slopes for fungus and density were estimated per species

Overall, mortality during the course of the experiment was low (Figure 1). Of the original 990 seedlings not exposed to fungicide, 920 survived (92.9%), whereas of the 990 fungicide treated seedlings, 943 survived (95.3%). Planting density and fungicide treatment all affected seedling mortality at both the species and overall community‐level, with greater survival of seedlings planted at low density and receiving fungicide treatments (Figure 2). The effects of fungicide application and planting density were present for all species. However, effects were relatively weak across species, with Bayesian CI for parameter estimates of fungicide application and planting density intersecting 0 for all species (Figure 3 and Table S3). Variation in the strength of treatment effects among species corresponds to variation in species daily death rate (Figure 2), with *S. pauciflora* exhibiting the greatest difference between high‐density fungicide treatments and having the greatest parameter estimate of fungicide application (Figure 3).

### 3.2 Relationships between local tree abundance and the strength of C‐NDD effects

**Figure 4:**
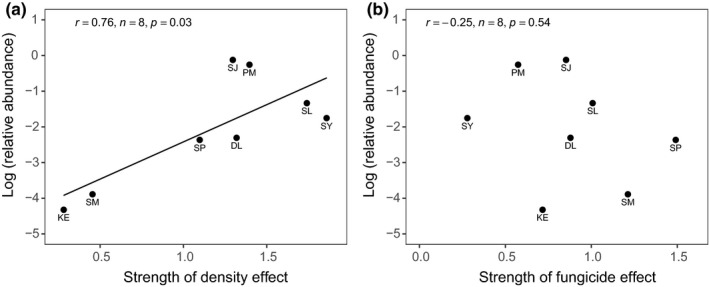
Relationships of variation in the strength of (a) planting density effects and (b) fungicide application effects on seedling survival with local adult tree abundance (>10 cm d.b.h). The strength of treatment effects represents regression coefficients estimated using generalized linear mixed models with binomial distribution and log‐link function within a hierarchical Bayesian framework. Pearson correlation coefficients (*r*) and *p*‐values (*p*) given in text

The strength of species‐level planting density effects on seedling survival was positively correlated with local adult abundances (*F* value = 8.01, *p* = .029, *df* = 1,7), with species exhibiting stronger density effects being more abundant in the surrounding forests of Danum Valley (Figure 4a). In comparison, the strength of fungicide application effects on seedling survival was uncorrelated with local adult abundances (Figure 3b; *F* value = 0.41, *p* = .546, *df* = 1,7).

### 3.3 Seedling height, stem diameter and leaf damage score

Mean seedling height, stem diameter, and leaf damage score, across all seedlings, were affected by species identity but not density and fungicide treatment (Table S4). When modeling species separately, only four of the eight species were affected by either density or fungicide treatments (*S. symingtonii, S. leprosula, D. lanceolata,* and *K. excelsa*).

Planting density, fungicide application, and the interaction between the two, each affected seedling height of a different single species (Koompasia excelsa, Shorea symingtonii and Shorea leprosula, respectively), with significantly taller individuals grown in low density pots or those treated with fungicide (Figure S1,Table S5). Seedling stem diameter was also affected by fungicide treatment and density in three of the eight species (Figure S2). *S. symintonii* was the only species to be affected by both fungicide and density treatments, with greater mean stem diameter of seedlings planted at low density and sprayed with fungicide (Table S6). Leaf damage score of seedlings was affected by fungicide application but not density, with two species exhibiting significantly greater leaf damage scores when treated with fungicide (Figure S3; Table S7). Additionally, only a single species exhibited interactive effects between treatments, with the effects of fungicide application on *S. leprosula* seedling height (*F* value = 22.4, *p* < .001, *df* = 1) and leaf damage score (*F* value = 12.59, *p* < .001, *df* = 1) varying with planting density.

## 4 DISCUSSION

Assessing the contributions of plant pathogens to C‐NDD across species and systems represents a key step in understanding their role in maintaining and structuring plant communities (Bagchi et al., 2010; Bell et al., 2006; Comita et al., 2014; Swinfield et al., 2012). Our results demonstrate weak overall C‐NDD (Figure 1), with varied but limited effects of fungal exclusion and planting density on seedling mortality across species. Manipulating both seedling density and pathogen infection, our experiment represents one of only a handful of studies assessing Janzen‐Connell mechanisms within SE Asia's mast‐fruiting forests (Blundell & Peart, 2004; Comita et al., 2014; Kurten & Carson, 2015) and the first of such studies to provide direct evidence of the role of fungal pathogens in mediating C‐NDD. Our findings indicate that, alone, fungal pathogens are likely insufficient to elicit significant diversifying effects on the mortality of seedlings. Despite strong evidence of plant pathogens as a powerful demographic force governing seedling mortality in neotropical forests (Bell et al., 2006; Sarmiento et al., 2017), this pattern is not ubiquitous, at least for our focal species, and appears comparatively less important in this mast‐fruiting system.

Previous studies of C‐NDD seedling mortality are rare in the Asian tropics, leading to the reporting of limited differences in the strength of density‐dependence between tropical regions (Carson et al., 2008; Comita et al., 2014). Our results are consistent with predictions that density‐dependent effects are substantially weaker among mast‐fruiting systems (Bagchi et al., 2011; Comita et al., 2014; Janzen, 1970), and starkly contrast with the consistent, strong pathogen‐induced C‐NDD seedling mortality observed in neotropical studies (Bagchi et al., 2010; Bell et al., 2006; Swinfield et al., 2012). It seems likely that ecological differences between masting and annually fruiting systems result in variation in the strength of C‐NDD between region, at least in terms of the contributions of fungal pathogens.

Recent studies have attributed variation in the strength of C‐NDD to differences between species and their associated traits (Jia et al., 2020; Kobe & Vriesendorp, 2011). Different mycorrhizal associations vary in their ability to protect hosts from soil pathogens, with ectomycorrhizal (ECM) associations potentially reducing susceptibility of seedlings to fungal pathogens via positive plant–soil feedbacks (PSFs), including the facilitation of nutrients and providing direct protection from antagonistic pathogens (Laliberté et al., 2015). Temperate studies from the New‐ and Old‐World have found contrasting effects of mycorrhizal associations on the strength of C‐NDD (Bennett et al., 2017; Jia et al., 2020), with ECM‐associated species experiencing greater negative effects of C‐NDD in comparison to AM species in a temperate Asian forest but the reverse across North American forests.

In the tropics, ECM species experience positive plant–soil feedback on seedling growth in Malaysian Borneo (Segnitz et al., 2020). We would expect to find strong C‐NDD under high‐density treatments, thus, our findings of limited density‐dependent seedling mortality across all six of our focal ECM species at first appear consistent with potential positive PSFs from ECM associations. However, the strength of C‐NDD was similarly weak across species, irrespective of mycorrhizal association suggesting that, at least for our focal species, mycorrhizal association did not dramatically effect seedling mortality. This dichotomy is likely due to mycorrhizal associations commonly being established later than the seedling stage and, thus ECM fungi are unable to convey recruitment advantages until much later in development (Jia et al., 2020). As tropical recruitment is highly constrained by seedling mortality during the seed‐to‐seedling transition (Bell et al., 2006; Harms et al., 2000), later establishment of fungal associations would greatly limit their role in mediating the strength C‐NDD, in comparison to other systems.

Ectomycorrhizal‐associated species are far more prevalent in Borneo and other SE Asian forests, compared to the neotropics, including all ~270 Bornean Dipterocarp species (Smith et al., 2013). Variation in the prevalence of different mycorrhizal associations between forest systems may partly be responsible for driving patterns of fungal‐induced C‐NDD. However, such mechanisms directly oppose J‐C with positive PSFs reducing the capacity for species coexistence by removing “rare species advantage” (Stump & Comita, 2018).

Natural enemies are unlikely to enhance plant community diversity unless the functional form of density‐dependence is overcompensating (Freckleton & Lewis, 2006). C‐NDD can be regarded as overcompensating when the number of recruits declines at higher initial densities, maximizing the likelihood of replacement by heterospecifics. Although uncommon among plant communities (Freckleton & Watkinson, 2002), Bagchi et al. (2010) demonstrate strong overcompensating C‐NDD of *P. longicuspis* seedlings, resulting in complete mortality at the highest seedling densities. In contrast, we observed weak overall mortality at very high densities, suggesting that density‐dependence is unlikely to represent an overcompensating form. Our high‐density treatment was much higher than typically observed in natural seedling banks. Thus, we would have expected to see extremely high mortality if density‐dependence was overcompensating. Although other functional forms of C‐NDD can contribute to coexistence by limiting species population sizes (Muller‐Landau & Alder, 2007), the likelihood of significant diversifying effects is severely reduced and such mechanisms differ to that originally proposed by Janzen, 1970 (Janzen, 1970) and Connell, 1971 (Connell, 1971).

Previous studies have demonstrated that locally abundant species are less susceptible to pathogen infection, experiencing weaker C‐NDD relative to locally rare species (Johnson et al., 2012; Mangan et al., 2010). The observed strength of C‐NDD may thus be underestimated due to selection of locally common species with sufficient availability of seeds for experimental manipulation (Comita et al., 2014). Instead, we find the strength of density effects on seedling survival was positively correlated with species’ relative abundances, with more common species experiencing greater density effects (Figure 4a). Our approach avoids many of the pitfalls of previous C‐NDD studies that often cause underestimation of the strength of C‐NDD, particularly for more abundant species (Detto et al., 2019). Moreover, seedling mortality during the experiment was low across species, irrespective of local abundance and fungicide treatment, suggesting that the strength of C‐NDD in this system was potentially weak.

Although limited to species with sufficient seed availability, our focal species represent 40% of all species included in studies experimentally testing the effects of fungal pathogens on C‐NDD across the tropics and is the largest set of species assessed from a single site (Table S1). Although we are reticent in suggesting our results depict community‐level contributions of plant pathogens to C‐NDD, we find little evidence to suggest fungal pathogens play a considerable role in determining seedling recruitment patterns across a range of masting species. Further studies are required to test the generality of our results for a greater number of species, encompassing a broader range of plant traits, and decipher the complex drivers regulating fungal–pathogen contributions to C‐NDD (Jia et al., 2020).

The limited contribution of plant–pathogens to early seedling mortality measured across our species suggests that other natural enemies are, at least partly, responsible for generating mortality and structuring the community. Previous studies have repeatedly emphasized large mammalian vertebrates as important drivers of both seed and seedling mortality in the tropics (Rosin et al., 2017), mediating abundances and densities of young stems (Harrison et al., 2013; Kurten & Carson, 2015, Villar et al. 2020) and causing the majority of mortality (Bagchi et al., 2011; Itoh et al., 1995). For example, four studies in Malaysian Borneo attribute up to 70%–86% of seed and young‐seedling mortality to herbivore browsing and seed predation (Bagchi et al., 2011; Granados et al., 2017; Hautier et al., 2010; Pillay et al., 2018). Although small, host‐specific herbivores can contribute to C‐NDD in isolation (Hautier et al., 2010), it remains unclear whether complete herbivore communities, including generalists, are capable of promoting plant diversity, either by mediating C‐NDD or by controlling populations of competitively dominant species (Kurten & Carson, 2015). Limited but host‐specific fungal‐induced C‐NDD mortality, in addition to generalist vertebrate seedling mortality, could lead to overcompensating forms of density‐dependence sufficient to enhance plant community diversity. However, comparative field studies are needed to test this prediction, manipulating pathogen abundance, planting density, and vertebrate seed predation and herbivory. Such work could reveal the roles of different groups of natural enemies in collectively mediating density‐dependent mortality in mast fruiting systems.

Seedlings experienced below average rainfall during the first month of the experiment. Drier conditions may have contributed to weaker recorded C‐NDD, as increased moisture availability can enhance transmission of both above‐ and below‐ground soil pathogens (Dorrance et al., 2003; Rossi & Caffi, 2012), with reduced precipitation limiting their ability to cause early mortality (Milici et al., 2020). Swinfield *et al*. found increased frequency of watering resulted in significantly greater pathogen‐induced seedling mortality, although even the most infrequently watered seedlings experienced substantially greater mortality than observed in our study. Moreover, previous studies from our field site demonstrate similarly low mortality of young seedlings despite frequent watering, including many of our chosen species (O’Brien et al., 2015, 2017), suggesting that limited mortality likely reflects lower overall susceptibility of our seedling species to pathogens.

**Figure 5:**
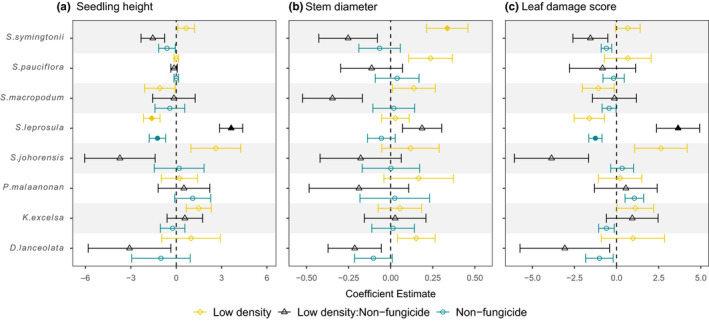
Model coefficients of the effects of planting density (high or low), fungicide treatment (+ or −) and the interaction between density and fungicide on; (a) mean seedling height, (b) stem diameter, and (c) leaf damage score for eight lowland tree species. Model coefficients estimated using linear and analysis of variance models. Filled points represent significant terms (*p* < .05) and error bars represent ± 1 *SE*

We observed an array of complex seedling growth responses to density and fungicide treatments (Figure 5; Figures S1, S2 and S3), with the effects of density and fungal pathogens on seedling growth being diverse and highly species specific. However, for the majority of species, seedling mortality rates were unaffected by density‐dependence in the absence of fungal pathogens, irrespective of effects on growth. This trend mirrors that of similar studies (Bagchi et al., 2010; Bell et al., 2006), suggesting that competition between conspecific seedlings is not a major contributor to early mortality in this tropical forests (Wright, 2002). However, growth advantages secured early in seedling development, potentially in response to plant pathogens, could also benefit long‐term survival by enabling larger individuals to outcompete their peers for limited resources such as light and space.

By focusing on the fate of seedlings immediately following germination, our results may not represent other life stages. For example, conditions beneficial to seed germination might coincide with substantial pathogen‐induced seed mortality by facilitating spread of infection. However, our treatments represent high but naturally occurring seedling densities, suggesting seed mortality attributed to fungal pathogens does not tightly limit seedling density. Furthermore, the proportion of seed mortality caused by pathogens is likely to be inconsequential given the majority of seeds are consumed by vertebrate seed predators during masting episodes (Bagchi et al., 2011; Granados et al., 2017). As with many short‐term manipulative studies, we do not track seedling survival past the seed‐to‐seedling transition. Longer‐term studies are required to test whether patterns of seedling dynamics structure adult community composition. This is particularly relevant in human‐modified forests where disturbances impact individuals long after their recruitment (Caughlin et al., 2014; Kurten & Carson, 2015).

In summary, we find evidence of fungal‐driven C‐NDD, with variation in the susceptibility of tree species to pathogen attack consistent with host‐specificity of pathogens. However, overall contributions of fungal pathogens to seedling mortality are low and resulting C‐NDD is unlikely to represent overcompensating forms. Thus, in the absence of other natural enemies, fungal pathogens appear insufficient in promoting the diversity of mast‐fruiting plant communities. Our study highlights the need to assess multiple species and regions, serving as an example of how the bias in tropical understanding from the neotropics can lead to commonly held beliefs that are not supported in other tropical systems. Further study is required to quantify the combined and separate roles of different natural enemies in driving patterns of seedling mortality and decipher how tree species traits, including fungal association, impact such patterns, and reveal how plant communities are maintained and structured within this globally important forest type.

## CONFLICT OF INTEREST

We declare no competing interests.

## AUTHOR CONTRIBUTION

**Patrick George Cannon:** Conceptualization (equal); Data curation (lead); Formal analysis (lead); Funding acquisition (supporting); Investigation (equal); Methodology (equal); Project administration (equal); Visualization (lead); Writing‐original draft (lead). **Michael J O'Brien:** Conceptualization (equal); Data curation (supporting); Formal analysis (supporting); Funding acquisition (equal); Methodology (equal); Project administration (equal); Resources (equal); Supervision (supporting); Writing‐review & editing (supporting). **Kalsum Mohd Yusah:** Project administration (equal); Resources (equal); Writing‐review & editing (supporting). **David P Edwards:** Funding acquisition (equal); Supervision (supporting); Writing‐review & editing (supporting). **Rob Freckleton:** Conceptualization (equal); Formal analysis (supporting); Funding acquisition (equal); Methodology (equal); Supervision (lead); Writing‐review & editing (supporting).

## Supporting information

Table S1‐S7 Fig S1‐S3

## Data Availability

Seedling leaf damage score, diameter growth, height, and mortality data: Dryad https://doi.org/10.5061/dryad.xksn02vdh .
