# Supplementary material for: Limited contributions of plant pathogens to density‐dependent seedling mortality of mast fruiting Bornean trees
Source: Ecol Evol. 2020 Oct 25;10(23):13154–64. doi: 10.1002/ece3.6906 (PMC7713929; doi:10.1002/ece3.6906)
Supplement: Supplementary file 1 — Table S1‐S7 Fig S1‐S3 [file ECE3-10-13154-s001.docx]

**Supporting Information:**

**Limited contributions of plant pathogens to density-dependent seedling mortality in mast fruiting Bornean trees**

Containing:

Table S1: Studies manipulating densities of tropical tree seeds and seedlings.

Table S2: Description of the eight lowland tree species used in the experiment.

Table S3: Overall and species-level effects of density and fungicide treatment on seedling mortality.

Table S4: Summary table of fitted models for seedling height, stem diameter and leaf damage score.

Table S5: Analysis of variance table of individual species linear regressions for seedling height.

Fig S1: Species-level density and fungicide effects on seedling height.

Table S6: Analysis of variance table of individual species linear regressions for seedling stem diameter.

Fig S2: Species-level density and fungicide effects on seedling stem diameter.

Table S7: Analysis of variance table of individual species linear regressions for leaf damage score.

Fig S3: Species-level density and fungicide effects on leaf damage score.

**Table S1**. Tropical studies manipulating seed or seedling densities of trees.

| **Region** | **Location** | **Life stage** | **Natural enemy tested** | **No. of species** | **Species** | **Study** |
| --- | --- | --- | --- | --- | --- | --- |
| Neotropics | Barro Colorado Island, Panama | seedling | none | 1 | *Cordia alliodora* | Downey *et al.*, 2018 |
|  | Western Brazil | seed | none | 1 | *Dypterix alata* | Ragusa-Netto, 2017 |
|  | Chiquibul Forest, Belize | seed, seedling | fungal pathogen | 5 | *Acacia polyphylla,*  *Cedrela odorata,*  *Cordia alliodora*  *Cryosophila stauracantha, Terminalia amazonia* | Gripenberg *et al.*, 2014 |
|  | Tinigua National Park, Colombia | seed | none | 1 | *Pachira quinata* | Castellanos and Stevenson, 2011 |
|  | Chiquibul Forest, Belize | seedling | fungal pathogen | 1 | *Pleradenophora longicuspis* | Bagchi *et al.*, 2010 |
|  | Pacaya-Samiria Reserve, Peru | seedling | none | 2 | *Garcinia macrophylla,*  *Xylopia micans* | Anderson J. T, 2008 |
|  | Chiquibul Forest, Belize | seedling | fungal pathogen | 1 | *Pleradenophora longicuspis* | Bell, Freckleton and Lewis, 2006 |
|  | Barro Colorado Island, Panama | seed, seedling | none | 1 | *Tachigalia versicolor* | Augspurger and Kitajima, 1992 |
|  | Mexico | seed | none | 1 | *Cymbopetalum baillonii* | Coates-Estrada and Estrada, 1988 |
|  | Atlantic lowlands, Costa Rica | seedling | none | 1 | *Dipteryx panamensis* | Clark and Clark, 1985 |
|  | Barro Colorado Island, Panama | seedling | none | 13 | *Platypodium elegans,*  *Lafoensia punicifolia,*  *Pseudobombax septenatum,*  *Triplaris cumingiana,*  *Luehea seemannii,*  *Tabebuia rosea,*  *Cordia alliodora,*  *Aspidosperma cruenata,*  *Terminalia oblonga,*  *Terminalia amazonica,*  *Ochroma pyramidale, Cochlospermum vitifolium,*  *Ceiba penta* | Augspurger and Kelly, 1984 |
| Afrotropics | North Eastern Gabon | seed | herbivores | 10 | *Pycnanthus angolensis,*  *Pentaclethra macrophylla, Pentaclethra eetveldeana, Cylicodiscus gabunensis, Pseudospondias microcarpa, Diospyros crassiflora, Scorodophloeus zenkeri, Piptadeniastrum africanum, Dacryodes buettneri,*  *Pterocarpus soyauxii* | Rosin and Poulsen, 2018 |
|  | Republic of Congo | seed, seedling | herbivores | 4 | *Pancovia laurentii,*  *Staudtia kamerunensis, Manilkara mabokeensis, Myrianthus arboreus, Entandophragma utile* | Clark, Poulsen and Levey, 2012 |
|  | Korup National Park, Cameroon | seedling | fungal pathogen | 2 | *Oubanguia alata, Microberlinia bisculcata* | Norghauer *et al.*, 2010 |
| Asian tropics | Western Ghats, India | seedling | fungal pathogen | 4 | *Toona ciliata, ﻿*  *Macaranga peltata, ﻿*  *Olea dioica, ﻿*  *Heritiera papilio* | Krishnadas and Comita, 2018 |
|  | Lambir Hills National Park, Malaysian Borneo | seed | none | 2 | *Shorea laxa,*  *Dipterocarpus tempehes* | Takeuchi and Nakashizuka, 2007 |
|  | North Queensland, Australia | seed | none | 1 | *Normanbya normanbyi* | Lott *et al.*, 1995 |

**Table S2.** Description of the eight lowland tree species used in the experiment. Relative abundance and stem densities calculated using data from the 50-Hectare Plot Project Danum Valley, part of the CTFS-ForestGEO network (Anderson-Teixeira *et al.*, 2015).

| Species | IUCN red list status | Relative abundance | Stems ha^-2^ | Total number of seedlings |
| --- | --- | --- | --- | --- |
| *Dryobalanops lanceolata* Burck * | LC | 0.10 | 5.26 | 264 |
| *Koompassia excelsa* (Becc.) Taub. ** | CD | 0.01 | 0.70 | 264 |
| *Parashorea malaanonan* Merr. * | LC | 0.77 | 40.28 | 242 |
| *Scaphium macropodum* Miq. ***** | LC | 0.02 | 1.08 | 242 |
| *Shorea johorensis* Foxw. * | CR | 0.88 | 46.70 | 242 |
| *Shorea leprosula* Miq. * | NT | 0.26 | 13.90 | 286 |
| *Shorea pauciflora* King* | EN | 0.09 | 4.96 | 264 |
| *Shorea symingtonii* G.H.S.Wood * | VU | 0.17 | 9.14 | 176 |
| Tree species family: ** Dipterocarpaceae; ** Leguminosae; *** Malvaceae.* | | | | |

**Table S3.** Overall and species-level effects of planting density (high or low), fungicide treatment (+ or -) and the interaction between density and fungicide on seedling mortality. Coefficients estimated using generalized linear mixed models with binomial distribution and log-link function within a hierarchical Bayesian framework allowing for estimation of coefficients despite no mortality recorded within some pots. Random uncorrelated slopes for fungus and density were estimated per species.

| **Term** | **Estimate** | **Error** | **CI 97.5%** | **CI 97.5%** |
| --- | --- | --- | --- | --- |
| **Across species** |  |  |  |  |
| Intercept | -2.87 | 2.21 | -6.98 | 1.73 |
| Density | -1.27 | 2.12 | -5.70 | 2.62 |
| Fungicide | 0.86 | 1.39 | -1.88 | 3.62 |
| Density x fungicide | -0.44 | 1.27 | -3.00 | 2.07 |
| ***Shorea pauciflora*** |  |  |  |  |
| Density | -1.20 | 2.17 | -5.82 | 2.73 |
| Fungicide | 1.48 | 1.38 | -1.22 | 4.25 |
| ***Shorea leprosula*** |  |  |  |  |
| Density | -1.88 | 2.26 | -6.69 | 2.20 |
| Fungicide | 1.01 | 1.40 | -1.71 | 3.84 |
| ***Shorea johorensis*** |  |  |  |  |
| Density | -1.39 | 2.16 | -5.93 | 2.56 |
| Fungicide | 0.83 | 1.40 | -1.93 | 3.60 |
| ***Shorea symingtonii*** |  |  |  |  |
| Density | -2.00 | 2.33 | -6.89 | 2.20 |
| Fungicide | 0.24 | 1.55 | -2.91 | 3.22 |
| ***Parashorea malaanonan*** |  |  |  |  |
| Density | -1.50 | 2.19 | -6.14 | 2.52 |
| Fungicide | 0.56 | 1.44 | -2.30 | 3.40 |
| ***Dryobalanops lanceolata*** |  |  |  |  |
| Density | -1.42 | 2.16 | -6.01 | 2.46 |
| Fungicide | 0.86 | 1.40 | -1.91 | 3.64 |
| ***Scaphium macropodum*** |  |  |  |  |
| Density | -0.51 | 2.06 | -4.91 | 3.28 |
| Fungicide | 1.18 | 1.41 | -1.59 | 3.96 |
| ***Koompasia excelsa*** |  |  |  |  |
| Density | -0.31 | 2.03 | -4.61 | 3.38 |
| Fungicide | 0.66 | 1.45 | -2.20 | 3.53 |

**Table S4.** Summary of analysis of variance tables of fitted models for seedling height, stem diameter and leaf damage score. Significant terms in bold.

| Model | Term | d.f. | Sum sq. | *F* value | *P* value |
| --- | --- | --- | --- | --- | --- |
| ***lm*** | ***Height*** |  |  |  |  |
|  | **Species** | **7** | **57746** | **336.74** | **0.000** |
|  | Density | 1 | 5 | 0.20 | 0.656 |
|  | Fungicide | 1 | 76 | 3.11 | 0.076 |
|  | Density x fungicide | 1 | 9 | 0.37 | 0.539 |
| ***lm*** | ***Stem diameter*** |  |  |  |  |
|  | **Species** | **7** | **328.1** | **122.80** | **0.000** |
|  | Density | 1 | 1.1 | 2.94 | 0.094 |
|  | Fungicide | 1 | 0.3 | 0.72 | 0.406 |
|  | Density x fungicide | 1 | 0.6 | 1.70 |  |
| ***lm*** | ***Leaf damage score*** |  |  |  |  |
|  | **Species** | **7** | **186.6** | **10.64** | **0.000** |
|  | Density | 1 | 1.2 | 0.46 | 0.498 |
|  | Fungicide | 1 | 0.7 | 0.28 | 0.596 |
|  | Density x fungicide | 1 | 1.3 | 0.51 | 0.480 |

**Table S5.** Analysis of variance table of individual species linear regressions for seedling height. Significant terms in bold.

| Term | d.f. | Sums sq | *F* value | *P* value |
| --- | --- | --- | --- | --- |
| ***Shorea pauciflora*** |  |  |  |  |
| Density | 1 | 0.06 | 0.48 | 0.490 |
| Fungicide | 1 | 0.06 | 0.49 | 0.488 |
| Density x fungicide | 1 | 0.06 | 0.47 | 0.496 |
| Residuals | 44 | 5.82 |  |  |
| ***Shorea leprosula*** |  |  |  |  |
| Density | 1 | 0.52 | 0.27 | 0.604 |
| Fungicide | 1 | 4.31 | 2.25 | 0.140 |
| **Density x fungicide** | **1** | **42.91** | **22.44** | **0.000** |
| Residuals | 48 | 91.80 |  |  |
| ***Shorea johorensis*** |  |  |  |  |
| Density | 1 | 6.26 | 0.42 | 0.521 |
| Fungicide | 1 | 30.79 | 2.06 | 0.159 |
| Density x fungicide | 1 | 38.19 | 2.55 | 0.118 |
| Residuals | 40 | 597.78 |  |  |
| ***Shorea symingtonii*** |  |  |  |  |
| Density | 1 | 0.11 | 0.09 | 0.762 |
| **Fungicide** | **1** | **15.40** | **12.70** | **0.001** |
| Density x fungicide | 1 | 4.80 | 4.80 | 0.056 |
| Residuals | 28 | 33.95 | 3.96 |  |
| ***Parashorea malaanonan*** |  |  |  |  |
| Density | 1 | 1.99 | 0.26 | 0.616 |
| Fungicide | 1 | 19.24 | 2.46 | 0.125 |
| Density x fungicide | 1 | 0.69 | 0.09 | 0.767 |
| Residuals | 39 | 305.01 |  |  |
| ***Dryobalanops lanceolata*** |  |  |  |  |
| Density | 1 | 3.84 | 0.17 | 0.681 |
| Fungicide | 1 | 78.37 | 3.49 | 0.068 |
| Density x fungicide | 1 | 28.62 | 1.28 | 0.265 |
| Residuals | 44 |  |  |  |
| ***Scaphium macropodum*** |  |  |  |  |
| Density | 1 | 14.80 | 2.78 | 0.104 |
| Fungicide | 1 | 2.66 | 0.50 | 0.484 |
| Density x fungicide | 1 | 0.06 | 0.01 | 0.914 |
| Residuals | 39 | 207.62 |  |  |
| ***Koompasia excelsa*** |  |  |  |  |
| **Density** | **1** | **36.18** | **9.14** | **0.004** |
| Fungicide | 1 | 0.01 | 0.00 | 0.959 |
| Density x fungicide | 1 | 0.93 | 0.23 | 0.631 |
| Residuals | 42 | 166.27 |  |  |


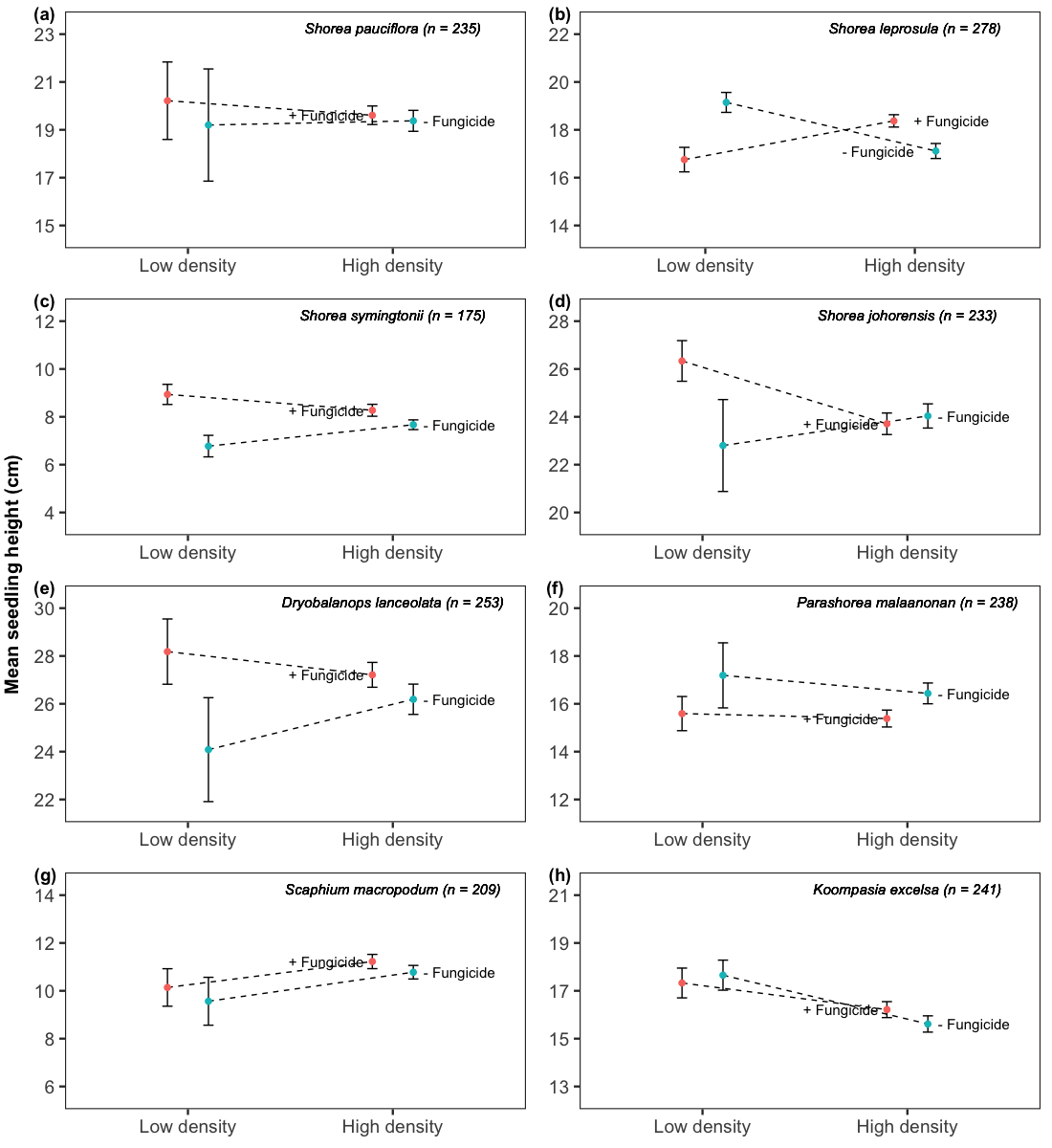


**Figure S1.** Mean seedling height (cm) of 1863 seedlings surviving 60 days after germination between pots planted at high and low density and treated with (+) or without (-) fungicide for each species: (**a**) *Shorea pauciflora*, (**b**) *Shorea leprosula*, (**c**) *Shorea symingtonii*, (**d**) *Shorea johorensis*, (**e**) *Dryobalanops lanceolata*, (**f**) *Parashorea malaanonan*, (**g**) *Scaphium macropodum*, and (**h**) *Koompasia exelsa*. Data points represent means across replicates and error bars denote standard error.

**Table S6.** Analysis of variance table of individual species linear regressions for seedling stem diameter. Significant terms in bold.

| Term | d.f. | Sums sq | *F* value | *P* value |
| --- | --- | --- | --- | --- |
| ***Shorea pauciflora*** |  |  |  |  |
| Density | 1 | 0.39 | 3.84 | 0.056 |
| Fungicide | 1 | 0.00 | 0.04 | 0.844 |
| Density x fungicide | 1 | 0.04 | 0.38 | 0.541 |
| Residuals | 44 | 4.46 |  |  |
| ***Shorea leprosula*** |  |  |  |  |
| **Density** | **1** | **0.19** | **4.35** | **0.042** |
| Fungicide | 1 | 0.02 | 0.42 | 0.518 |
| Density x fungicide | 1 | 0.11 | 2.57 | 0.115 |
| Residuals | 48 | 2.11 |  |  |
| ***Shorea johorensis*** |  |  |  |  |
| Density | 1 | 0.00 | 0.05 | 0.817 |
| Fungicide | 1 | 0.09 | 0.53 | 0.470 |
| Density x fungicide | 1 | 0.09 | 0.55 | 0.463 |
| Residuals | 40 | 6.41 |  |  |
| ***Shorea symingtonii*** |  |  |  |  |
| **Density** | **1** | **0.35** | **5.81** | **0.023** |
| **Fungicide** | **1** | **0.30** | **4.90** | **0.035** |
| Density x fungicide | 1 | 0.13 | 2.11 | 0.157 |
| Residuals | 28 | 1.70 |  |  |
| ***Parashorea malaanonan*** |  |  |  |  |
| Density | 1 | 0.06 | 0.26 | 0.611 |
| Fungicide | 1 | 0.05 | 0.21 | 0.649 |
| Density x fungicide | 1 | 0.10 | 0.41 | 0.528 |
| Residuals | 39 | 9.15 |  |  |
| ***Dryobalanops lanceolata*** |  |  |  |  |
| Density | 1 | 0.02 | 0.32 | 0.573 |
| **Fungicide** | **1** | **0.53** | **7.02** | **0.011** |
| Density x fungicide | 1 | 0.14 | 1.82 | 0.184 |
| Residuals | 44 | 3.30 |  |  |
| ***Scaphium macropodum*** |  |  |  |  |
| Density | 1 | 0.02 | 0.24 | 0.629 |
| Fungicide | 1 | 0.24 | 2.87 | 0.098 |
| Density x fungicide | 1 | 0.32 | 3.79 | 0.059 |
| Residuals | 39 | 3.31 |  |  |
| ***Koompasia excelsa*** |  |  |  |  |
| Density | 1 | 0.05 | 0.54 | 0.465 |
| Fungicide | 1 | 0.01 | 0.09 | 0.772 |
| Density x fungicide | 1 | 0.00 | 0.02 | 0.886 |
| Residuals | 42 | 4.03 |  |  |


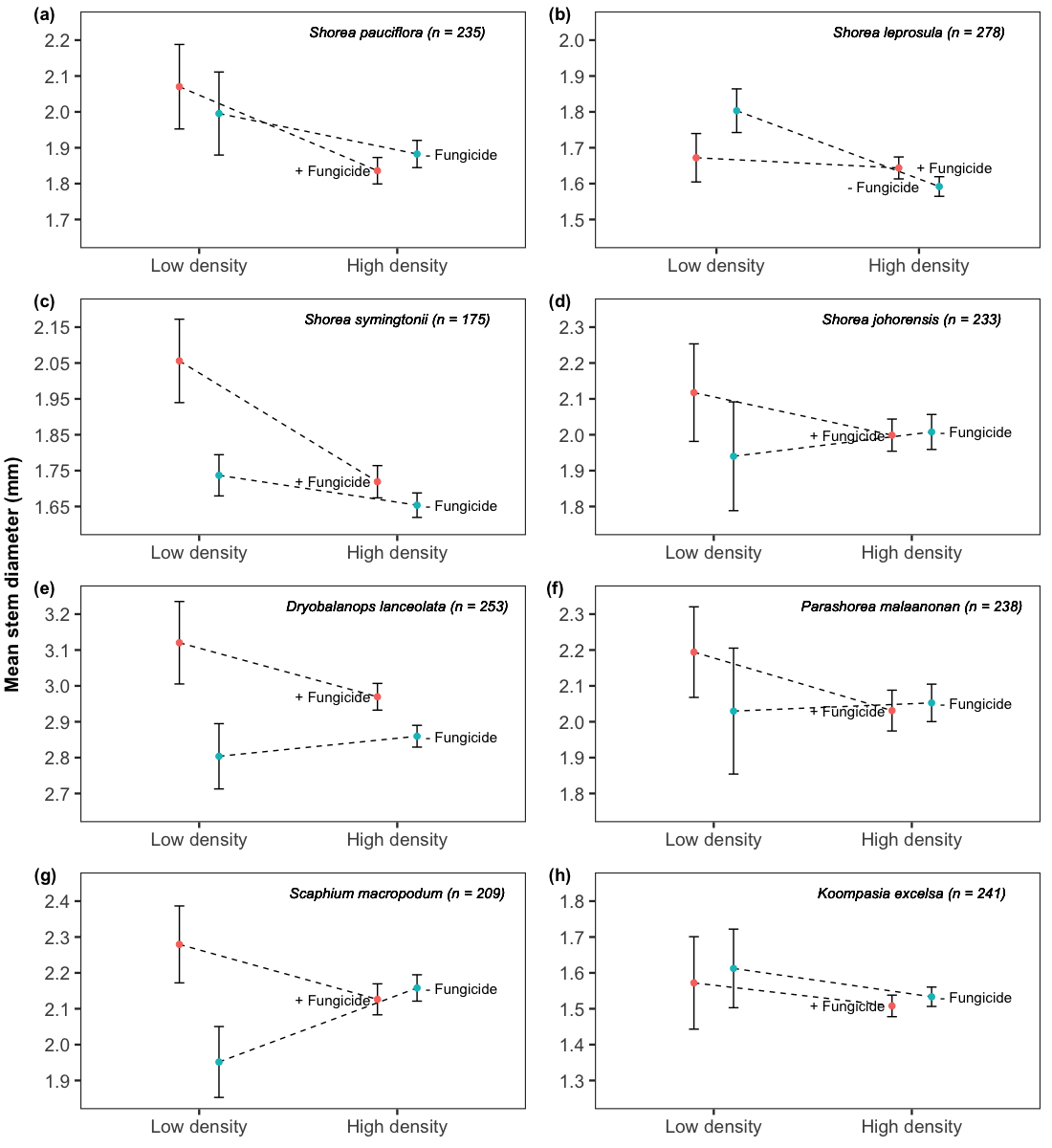


**Figure S2.** Mean stem diameter (mm) of 1863 seedlings surviving 60 days after germination between pots planted at high and low density and treated with (+) or without (-) fungicide for each species: (**a**) *Shorea pauciflora*, (**b**) *Shorea leprosula*, (**c**) *Shorea symingtonii*, (**d**) *Shorea johorensis*, (**e**) *Dryobalanops lanceolata*, (**f**) *Parashorea malaanonan*, (**g**) *Scaphium macropodum*, and (**h**) *Koompasia exelsa*. Data points represent means across replicates and error bars denote standard error.

**Table S7.** Individual species analysis of variance table for seedling leaf damage score. Significant terms in bold.

| Term | d.f. | Sums sq | *F* value | *P* value |
| --- | --- | --- | --- | --- |
| ***Shorea pauciflora*** |  |  |  |  |
| Density | 1 | 1.21 | 0.03 | 0.856 |
| Fungicide | 1 | 4.09 | 0.11 | 0.740 |
| Density x fungicide | 1 | 3.78 | 0.10 | 0.749 |
| Residuals | 44 | 1612.1 |  |  |
| ***Shorea leprosula*** |  |  |  |  |
| Density | 1 | 0.86 | 0.14 | 0.712 |
| **Fungicide** | **1** | **58.46** | **9.37** | **0.004** |
| **Density x fungicide** | **1** | **78.55** | **12.59** | **0.000** |
| Residuals | 48 | 299.46 |  |  |
| ***Shorea johorensis*** |  |  |  |  |
| Density | 1 | 9.67 | 0.26 | 0.613 |
| Fungicide | 1 | 0.08 | 0.00 | 0.963 |
| Density x fungicide | 1 | 74.37 | 2.00 | 0.166 |
| Residuals | 40 | 37.28 |  |  |
| ***Shorea symingtonii*** |  |  |  |  |
| Density | 1 | 0.19 | 0.04 | 0.852 |
| **Fungicide** | **1** | **24.42** | **4.63** | **0.040** |
| Density x fungicide | 1 | 8.82 | 1.67 | 0.207 |
| Residuals | 28 | 147.79 |  |  |
| ***Parashorea malaanonan*** |  |  |  |  |
| Density | 1 | 3.78 | 0.14 | 0.708 |
| Fungicide | 1 | 71.97 | 2.70 | 0.108 |
| Density x fungicide | 1 | 1.43 | 0.05 | 0.818 |
| Residuals | 39 | 26.66 |  |  |
| ***Dryobalanops lanceolata*** |  |  |  |  |
| Density | 1 | 7.06 | 0.15 | 0.696 |
| Fungicide | 1 | 109.18 | 2.39 | 0.129 |
| Density x fungicide | 1 | 51.46 | 1.13 | 0.294 |
| Residuals | 44 | 45.70 |  |  |
| ***Scaphium macropodum*** |  |  |  |  |
| Density | 1 | 25.87 | 1.92 | 0.174 |
| Fungicide | 1 | 11.03 | 0.82 | 0.371 |
| Density x fungicide | 1 | 0.08 | 0.01 | 0.939 |
| Residuals | 39 | 13.49 |  |  |
| ***Koompasia excelsa*** |  |  |  |  |
| Density | 1 | 50.34 | 3.31 | 0.076 |
| Fungicide | 1 | 16.03 | 1.05 | 0.310 |
| Density x fungicide | 1 | 4.31 | 0.28 | 0.598 |
| Residuals | 42 | 15.21 |  |  |


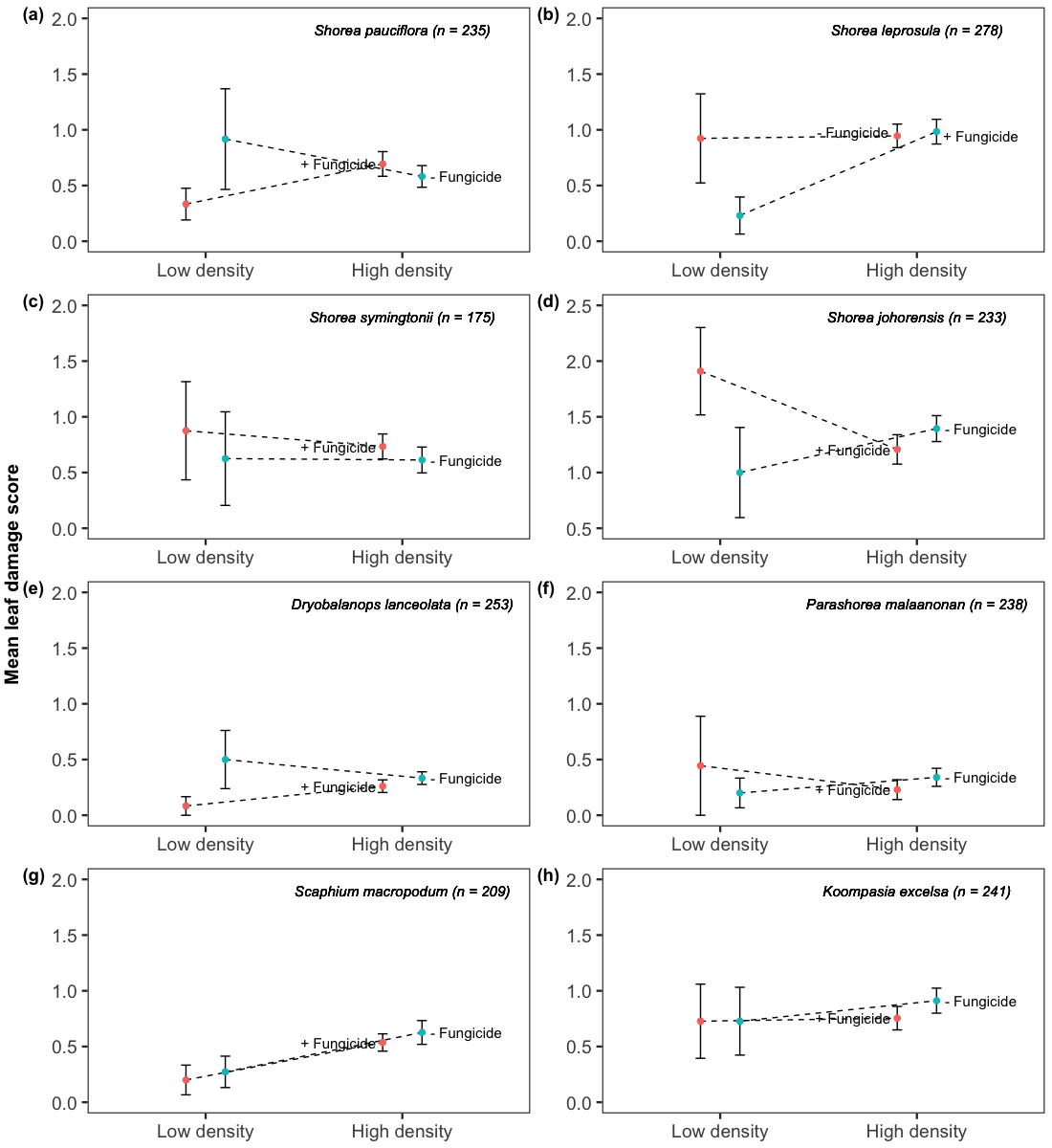


**Figure S3.** Mean leaf damage score (1 to 4 ordinal scale) of 1863 seedlings surviving 60 days after germination between pots planted at high and low density and treated with (+) or without (-) fungicide for each species: (**a**) *Shorea pauciflora*, (**b**) *Shorea leprosula*, (**c**) *Shorea symingtonii*, (**d**) *Shorea johorensis*, (**e**) *Dryobalanops lanceolata*, (**f**) *Parashorea malaanonan*, (**g**) *Scaphium macropodum*, and (**h**) *Koompasia exelsa*. Data points represent means across replicates and error bars denote standard error.

**References**

Anderson-Teixeira, K. J. *et al.* (2015) ‘CTFS-ForestGEO: A worldwide network monitoring forests in an era of global change’, *Global Change Biology*, 21(2), pp. 528–549. doi: 10.1111/gcb.12712.

Anderson J. T (2008) ‘Positive density dependence in seedlings of the neotropical tree species Garcinia macrophylla and Xylopia micans’, *Journal of Vegetation Science*, (June 2008), pp. 1–10. doi: 10.3170/2008-8-18488.

Augspurger, C. K. and Kelly, C. K. (1984) ‘Pathogen mortality of tropical tree seedlings: experimental studies of the effects of dispersal distance, seedling density, and light conditions’, *Acta Biotheoretica*, 61, p. 211 217. doi: 10.1007/BF00052146.

Augspurger, C. K. and Kitajima, K. (1992) ‘Experimental Studies of Seedling Recruitment from Contrasting Seed Distributions Author ( s ): Carol K . Augspurger and Kaoru Kitajima Published by : Wiley Stable URL : http://www.jstor.org/stable/1940675 REFERENCES Linked references are available on JSTO’, 73(4), pp. 1270–1284.

Bagchi, R. *et al.* (2010) ‘Testing the Janzen-Connell mechanism: Pathogens cause overcompensating density dependence in a tropical tree’, *Ecology Letters*, 13(10), pp. 1262–1269. doi: 10.1111/j.1461-0248.2010.01520.x.

Bell, T., Freckleton, R. P. and Lewis, O. T. (2006) ‘Plant pathogens drive density-dependent seedling mortality in a tropical tree’, *Ecology Letters*, 9(5), pp. 569–574. doi: 10.1111/j.1461-0248.2006.00905.x.

Castellanos, M. C. and Stevenson, P. R. (2011) ‘Phenology, seed dispersal and difficulties in natural recruitment of the canopy tree Pachira quinata (Malvaceae)’, *Revista de Biologia Tropical*, 59(2), pp. 921–933. doi: 10.15517/rbt.v0i0.3150.

Clark, C. J., Poulsen, J. R. and Levey, D. J. (2012) ‘Vertebrate herbivory impacts seedling recruitment more than niche partitioning or density-dependent mortality’, *Ecology*, 93(3), pp. 554–564. doi: 10.1890/11-0894.1.

Clark, D. B. and Clark, D. A. (1985) ‘Seedling Dynamics of a Tropical Tree : Impacts of Herbivory and Meristem Damage’, 66(6), pp. 1884–1892.

Coates-Estrada, R. and Estrada, A. (1988) ‘Frugivory and seed dispersal in cymhopetalum baillonii (annonaceae) at los tuxtlas, mexico’, *Journal of Tropical Ecology*, 4(2), pp. 157–172. doi: 10.1017/S0266467400002650.

Downey, H. *et al.* (2018) ‘Insect herbivory on seedlings of rainforest trees: Effects of density and distance of conspecific and heterospecific neighbors’, *Ecology and Evolution*, 8(24), pp. 12702–12711. doi: 10.1002/ece3.4698.

Gripenberg, S. *et al.* (2014) ‘Testing for enemy-mediated density-dependence in the mortality of seedlings: Field experiments with five Neotropical tree species’, *Oikos*, 123(2), pp. 185–193. doi: 10.1111/j.1600-0706.2013.00835.x.

Krishnadas, M. and Comita, L. S. (2018) ‘Influence of soil pathogens on early regeneration success of tropical trees varies between forest edge and interior’, *Oecologia*. Springer Berlin Heidelberg, 186(1), pp. 259–268. doi: 10.1007/s00442-017-4006-1.

Lott, R. H. *et al.* (1995) ‘Density-Dependent Seed Predation and Plant Dispersion of the Tropical Palm Normanbya normanbyi’, *Biotropica*, 27(1), p. 87. doi: 10.2307/2388906.

Norghauer, J. M. *et al.* (2010) ‘Do fungal pathogens drive density-dependent mortality in established seedlings of two dominant African rain-forest trees?’, *Journal of Tropical Ecology*, 26(3), pp. 293–301. doi: 10.1017/S0266467410000076.

Ragusa-Netto, J. (2017) ‘Seed removal of Dipteryx alata Vog. (Leguminosae: Faboidae) in the edge and interior of Cerrado’, *Brazilian Journal of Biology*, 77(4), pp. 752–761. doi: 10.1590/1519-6984.20715.

Rosin, C. and Poulsen, J. R. (2018) ‘Seed traits, not density or distance from parent, determine seed predation and establishment in an Afrotropical forest’, *Biotropica*, 50(6), pp. 881–888. doi: 10.1111/btp.12601.

Takeuchi, Y. and Nakashizuka, T. (2007) ‘Effect of distance and density on seed/seedling fate of two dipterocarp species’, *Forest Ecology and Management*, 247(1–3), pp. 167–174. doi: 10.1016/j.foreco.2007.04.028.
